# The Impact of Acute Rheumatic Fever Diagnosis on Rheumatic Heart Disease Severity

**DOI:** 10.5334/gh.1454

**Published:** 2025-08-29

**Authors:** Jacqueline Maree Williamson, Gillian Whalley, Simon Thornley, James Marangou, Peter Morris, Joshua R. Francis, Vicki Wade, Bo Remenyi

**Affiliations:** 1 Menzies School of Health Research, Charles Darwin University, Australia; 2 University of Otago, Dunedin, New Zealand; 3 University of Auckland, Auckland, New Zealand; 4 Menzies School of Health Research, Darwin and Cardiology Department and Royal Perth Hospital, Perth, Australia; 5 Menzies School of Health Research and Royal Darwin Hospital, Northern Territory, Australia; 6 Menzies School of Health Research, Darwin, Australia

**Keywords:** Rheumatic heart disease, rheumatic fever, echocardiography, diagnosis

## Abstract

**Background:**

Acute rheumatic fever (ARF) is the precursor to rheumatic heart disease (RHD) following Group A Streptococcal infection. However, many diagnoses of RHD are made in the absence of ARF history. We compared RHD severity between those with and those without a documented history of ARF.

**Methods:**

A retrospective audit of echocardiographic images determined RHD stage at diagnosis and at follow-up based on the 2023 WHF guidelines for the diagnosis of RHD.

Individuals aged ≤ 20 years from the Top End of the Northern Territory (NT) of Australia with RHD diagnosis between January 2012 and December 2021 were included.

Primary outcome was RHD stage at the time of diagnosis. Secondary outcomes were RHD stage progression or regression. Those with ARF and those with no ARF (noARF) were compared.

**Results:**

Study population (*n*) of 292 individuals with mean age 11.9 ± 3.8 years. At baseline, the ARF group had more Stage A RHD (28.6% versus 12.0%), while the noARF group had more Stage B (50.0% versus 38.0%), *p* = 0.009. There was no difference in advanced RHD (Stage C and D combined) between the groups (*p* = 0.440). Follow-up (median 46 months, IQR: 27–71 months) sample size was 230. Regression of RHD was greater in the ARF group (46% versus 28%, *p* = 0.014). No difference was found in stage progression (including to surgery), with 21% (32/156) in the ARF group and 15% (11/74) in the noARF group (*p* = 0.367).

**Conclusions:**

Individuals at all stages of RHD severity were detected amongst those with and without an accompanying diagnosis of ARF. Individuals with first RHD diagnosis accompanied by ARF were more likely to regress. These findings support echocardiographic screening in high-risk populations to detect early RHD that can be treated with secondary antibiotic prophylaxis. Further research is required to understand the reason for differences between the ARF and noARF groups.

## Introduction

Rheumatic heart disease (RHD) remains a significant health concern for many of Australia’s Aboriginal and Torres Strait Islander people. Acute rheumatic fever (ARF), associated with Group A streptococcal infection, precedes RHD, however many individuals are diagnosed with RHD through echocardiographic imaging without a known episode of ARF (1,2). These presumably ‘missed’ ARF episodes represent more than one third of Australian cases (3), and are detected through i) incidental finding of a murmur or other clinical feature; ii) presentation once clinical symptoms have developed; or iii) echocardiographic screening of asymptomatic people at risk of RHD.

It is not currently known whether RHD prognosis differs between those with a clinical ARF presentation and those without. A previous study from Fiji reported approximately 16% of cases detected through echocardiographic screening developed severe RHD without adequate secondary antibiotic prophylaxis (SAP) (4). Secondary antibiotic prophylaxis involves the regular administration of antibiotics (usually long-acting benzathine benzylpenicillin G via intra-muscular injection every 3–4 weeks) to prevent future streptococcal infection (5). If ARF is undiagnosed, the opportunity for early commencement of SAP is missed.

We hypothesised that patients without ARF presentation would have more significant RHD at the time of first diagnosis compared to those with one or multiple documented ARF episodes. This assumes that patients with an ARF diagnosis were prescribed SAP at an earlier stage of illness. Further, we investigated differences in prognosis between the groups, speculating that RHD progression and regression would be similar following RHD detection and commencement of SAP.

Our study was undertaken in the Northern Territory (NT) of Australia, where both ARF and RHD are notifiable diseases. The NT is a vast land positioned in the central north of Australia and is characterised by remote Aboriginal communities and homelands, many of which are hundreds of kilometres from tertiary referral centres. The NT is home to the majority of Australia’s RHD and ARF cases (3), with one large remote community reported to have the highest burden of RHD globally (6). We undertook an audit of RHD cases from the Top End of the NT and our aims were: i) to compare the RHD stages at diagnosis between those with and without a documented first episode of ARF; and ii) to compare the prognosis (in terms of progression or regression of RHD) between those with ARF and those without.

This novel investigation may have implications for echocardiographic screening for RHD by providing insight into RHD prognosis for those without any history of ARF episodes. This may improve our understanding of risk and management of children detected either through active case finding or routine clinical care.

## Methods

### Population

Children and young people (≤ 20 years) from the Top End of NT, Australia with an RHD diagnosis recorded in the NT RHD register between January 2012 and December 2021 were included. The NT RHD register records all compulsory notifications of both ARF and RHD made by medical practitioners. This includes cases of any severity (including the now defunct borderline RHD classification) detected through clinical presentation or screening. Those with congenital heart lesions or missing echocardiographic data at the time of RHD diagnosis were excluded. Those with follow-up echocardiograms were reviewed to evaluate RHD progression and regression.

Human research ethics approval was sought from Menzies School of Health Research with approval granted for this low-risk review of retrospectively captured clinical data (HREC: *2021–4146*).

### Data collection

Echocardiographic images had been acquired by accredited cardiac sonographers, paediatric cardiologists, or a paediatrician with experience in echocardiography for RHD. Studies were performed on a range of high-end ultrasound machines and portable devices with full-service capability. Original reports were generated by a single clinician (multiple reviewers, designated as Observer 1). Post-hoc review of all available echocardiographic studies and independent reporting was performed by a single operator (JW, Observer 2) who was blind to the original diagnostic report and to the ARF status at the time of image review. Images were reviewed offline (Synapse, Fujifilm), and diagnostic reports were used for cross reference and severity data when echocardiographic images were unavailable.

### Reporting of valvular lesions

Individual lesions involving mitral regurgitation (MR), mitral stenosis (MS), aortic regurgitation (AR), and aortic stenosis (AS) were assessed using American Society of Echocardiography guidelines (7,8), as per routine practice with further details in Supplement 1.

Lesion findings were compared with the original reports from Observer 1. If the findings between Observer 1 and Observer 2 differed by more than half one grade in severity, or if no clinical report was available, cases were reviewed by a third party (GW, Observer 3) who was aware of the findings of Observer 1 and Observer 2 but remained blind to the ARF status. Final grade was by consensus ≥ 2 observers. Severity of each lesion was graded as either none, physiological, mild, moderate, or severe.

### Baseline RHD stage

The presence of pathological mitral and/or aortic regurgitation with or without abnormal mitral and/or aortic valve features permit the diagnosis or RHD once other causes have been excluded (9). The specific valvular features consistent with RHD are detailed in Supplement 2. The 2023 WHF guidelines for the echocardiographic diagnosis of RHD (9) were applied retrospectively to diagnose and classify RHD (Table 1).

**Table 1:** Rheumatic heart disease stages (9).


Stage A

mild isolated pathological MR, or

mild isolated pathological AR.

**Stage B**

mild pathological MR and mild pathological AR, or

mild pathological MR with morphological abnormality/s* of the mitral valve, or

mild pathological AR with morphological abnormality/s* of the aortic valve.

**Stage C**

at least moderate MR, or

at least moderate AR, or

any mitral stenosis, or

any aortic stenosis.

**Stage D**

any of the Stage C lesions requiring surgical intervention**


*At least one morphological abnormality required if ≤ 20 years; at least two abnormalities required if >20 years. **The guidelines specify the presence of clinical complications including the need for surgical intervention constitutes Stage D RHD. In the Australian setting, surgery is an option for all those requiring it, and since this audit did not include information on clinical history, surgical events provided by the RHD register were used to indicate the presence of Stage D RHD in this cohort.

### Follow-up RHD stage

Patients with follow-up echocardiographic data > six months after RHD diagnosis (as of 31 December 2022) were included in the RHD progression and regression analysis. The most recent echocardiogram was included, giving the longest follow-up period for each individual. In those with Stage D RHD, follow-up was censured at the date of surgery. In addition to the Stage A, B, C, and D categories used at baseline, a category of ‘resolved/no RHD’ was included:

***Resolved/no RHD:*** Absence of echocardiographic features of RHD meeting the 2023 WHF RHD guidelines (9) at follow-up.

Changes in RHD stage were classified as follows:

***Progression:*** An advance in the RHD stage allocated at baseline. For example, Stage A progressing to Stage B, C, or D at follow-up, or Stage C progressing to Stage D (surgery).

***Regression:*** A reduction in the RHD stage allocated at baseline. For example, Stage A regressing to no RHD, or Stage C regressing to Stage A, B, or no RHD.

***Unchanged:*** RHD stage remaining the same at follow-up as observed at baseline.

### Stratification of ARF groups

The Australian ARF/RHD guidelines (5), which incorporate the modified Jones’ criteria (10), were used for the diagnosis of ARF. These criteria include major and minor criteria based on clinical presentation, echocardiographic and electrocardiographic findings, and blood results. All findings of possible, probable, or definite ARF as defined by the Australian guidelines (5) are reported to the RHD register. The NT RHD register data were used to determine the timing of ARF episodes and RHD diagnoses. Cases of possible, probable, or definite ARF at the time of RHD diagnosis (or within a six-month window) define the ARF group. Those without a documented history of ARF prior to, or within six months of, a diagnosis of RHD constitute the noARF group. The six-month window allowed for echocardiograms to be scheduled and completed.

### Statistical analysis

Echocardiographic findings were recorded in REDCap electronic data capture tool hosted at Menzies School of Health Research. R software (version 4.4.0) was used to detect association between baseline factors using t-test, chi square, or Fisher’s exact test (for counts <10). Statistical significance was defined using a threshold of 5% (p < 0.05).

### Data accessibility

The study data can be accessed by contacting the corresponding author.

## Results

**Figure 1:**
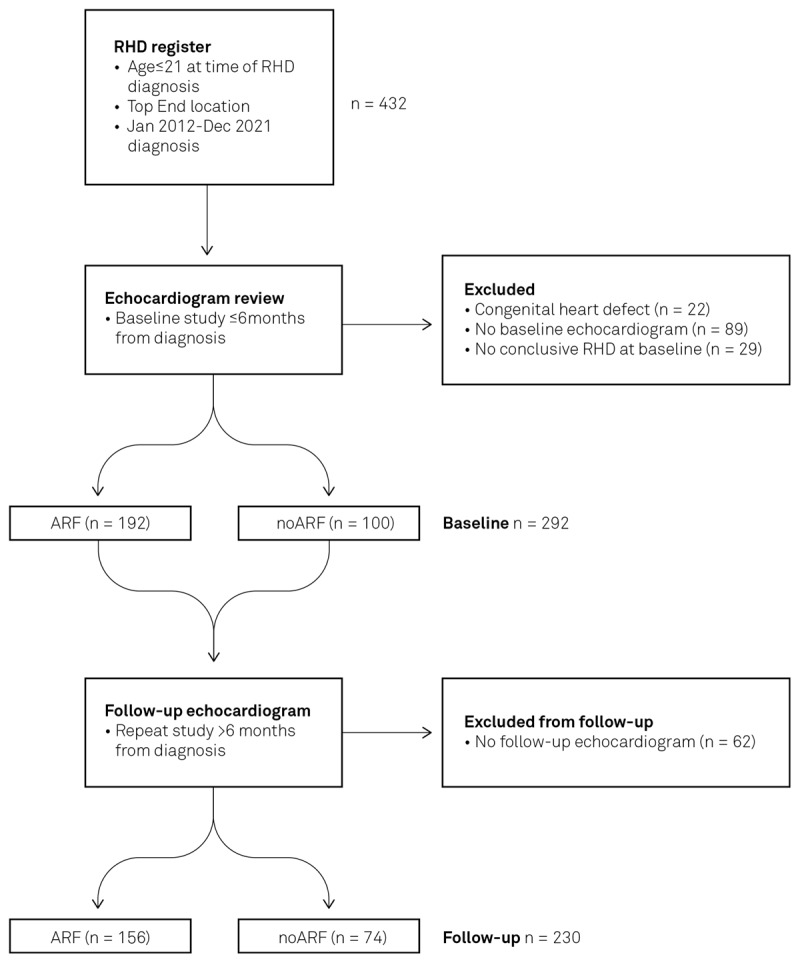
Patient inclusion and stratification process. Registry data identified individuals with RHD and ARF diagnoses. Echocardiographic images were reviewed to determine RHD stage at baseline and at follow-up.

Four hundred and thirty-two children met the initial inclusion criteria, with 22 children excluded due to the presence of a congenital heart lesion (including cases of atrial septal defect, ventricular septal defect, patent ductus arteriosus, bicuspid aortic valve, dilated coronary sinus, rhabdomyoma, or cleft mitral valve). A further 89 were excluded as echocardiographic data was unavailable within six months of RHD diagnosis. Twenty-nine were excluded due to re-classification to ‘no RHD’ upon post-hoc review by Observer 2, creating a baseline study cohort (*n*) of 292 patients (Figure 1). This included nine patients who met the contemporaneous 2012 WHF guideline criteria (11) for diagnosis of borderline RHD with morphological changes to the mitral valve without accompanying MR. These nine were excluded, as this finding is regarded as a normal variant in the updated 2023 WHF guidelines (9). Original diagnostic reports were used on occasions where echocardiographic images were unavailable (*n* = 21 for baseline studies and *n* = 7 at follow-up). Observer 3 was required to achieve consensus in 21% of cases where there was a difference in lesion classification between observers 1 and 2. Median follow-up length was 46 months (IQR: 27–71 months).

The study population was divided into two groups: those with clinical evidence of ARF at the time of RHD diagnosis, *n* = 192 (65.8%), and those without ARF history, *n* = 100 (34.2%). Two-thirds (126/192) of the ARF group were diagnosed with RHD within the first 24 hours of their index ARF presentation. A further 21% (*n* = 41) were diagnosed by day seven following ARF diagnosis; RHD diagnosis occurred within one month in 94% (181/192) and within three months in 99% (191/192). The final individual was diagnosed with RHD six months following ARF diagnosis. The majority of individuals in the ARF group had definite ARF at the time of RHD diagnosis, and 6.8% (13/192) had possible or probable ARF.

### Patient demographics

Mean age at the time of RHD diagnosis was 11.9 years (standard deviation (sd) = 3.8, range: 2–18 years). One hundred and sixty-one of the 192 patients (55.1%) were female, and 99% (290/292) were Aboriginal Australians.

Those in the ARF group were younger at the time of RHD diagnosis (mean 11.4 years) compared to the noARF group (mean 12.8 years; *p* = 0.002). There was a higher proportion of females (65%) in the noARF group compared to the ARF group (50%, *p* = 0.018; Table 2).

**Table 2:** Summary of patient data, RHD severity, and lesion type at baseline.


	ARF *n* = 192 *n* (%)*	No ARF *n* = 100 *n* (%)*	*P*-value^†^

Age at baseline (years; mean (sd))	11.4 (3.7)	12.8 (3.9)	0.002

Female	96 (50.0%)	65 (65.0%)	0.018

***RHD stages***			0.009

Early RHD – Stage A	55 (28.6%)	12 (12.0%)	

Early RHD – Stage B	73 (38.0%)	50 (50.0%)	

Advanced RHD – Stage C	55 (28.6%)	31 (31.0%)	

Advanced RHD – Stage D	9 (4.7%)	7 (7.0%)	

Total early RHD	128 (66.7%)	62 (62.0%)	0.440

Total advanced RHD	64 (33.3%)	38 (38.0%)	

***Lesion type***			

Pathological mitral regurgitation – all**	169 (88.0%)	88 (88.0%)	0.996

Pathological aortic regurgitation – all**	85 (44.3%)	39 (39%)	0.387

Isolated mitral regurgitation	40 (20.8%)	7 (7.0%)	0.002

Isolated aortic regurgitation	17 (8.9%)	6 (6.0%)	0.495

MR and AR (no abnormal morphology)	10 (5.2%)	3 (3.0%)	0.553

MR with abnormal morphology (+/– AV disease)	117 (60.9%)	76 (76.0%)	0.010

AR with abnormal morphology (+/– MV disease)	33 (17.2%)	20 (20.0%)	0.554

Any mitral stenosis	4 (2.1%)	3 (3.0%)	0.694

Any aortic stenosis	0 (0%)	1 (1.0%)	0.343


*Column percentage, unless otherwise specified. ^†^Fisher test, unless otherwise specified. **Includes pathological regurgitation in isolation and in combination with other valve lesions. AV, aortic valve; ARF, acute rheumatic fever; AR, aortic regurgitation; MR, mitral regurgitation; MV, mitral valve; RHD, rheumatic heart disease, sd, standard deviation.

### Baseline RHD stages

**Figure 2:**
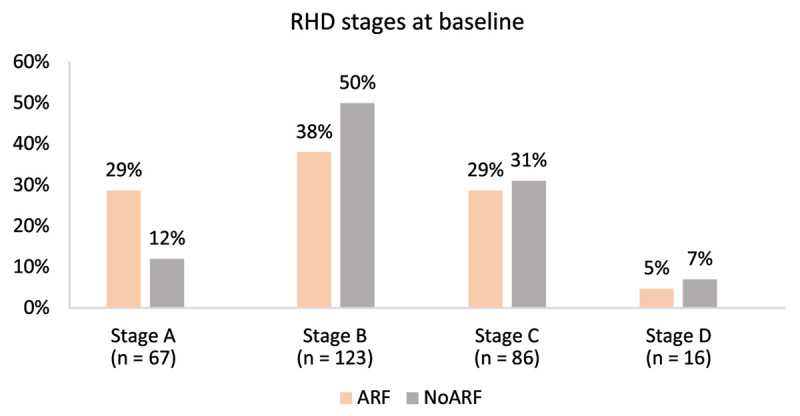
RHD stage at baseline echocardiogram stratified by the presence of ARF or no ARF.

The ARF group contained more individuals with Stage A RHD, while the noARF group contained more with Stage B (*p* = 0.009; Figure 2). When stages were dichotomised into early RHD (stage A and B) and advanced (stage C and D), there was little difference in the proportions between the groups with approximately two-thirds early RHD and one-third advanced RHD in each ARF status group (*p* = 0.440; Table 2).

### Lesion type at baseline

Pathological MR was the most common valve lesion, present in 88% of individuals in each group. There was a greater proportion of isolated pathological MR (Stage A) in the ARF group (20.8%) compared to 7.0% in the no ARF group (*p* = 0.002). By the same token, there were more cases of MR with morphological mitral valve abnormalities (Stage B or greater) in the noARF group (76.0%) compared to the ARF group (60.9%, *p* = 0.010, Table 2).

### Follow-up RHD stages

**Figure 3:**
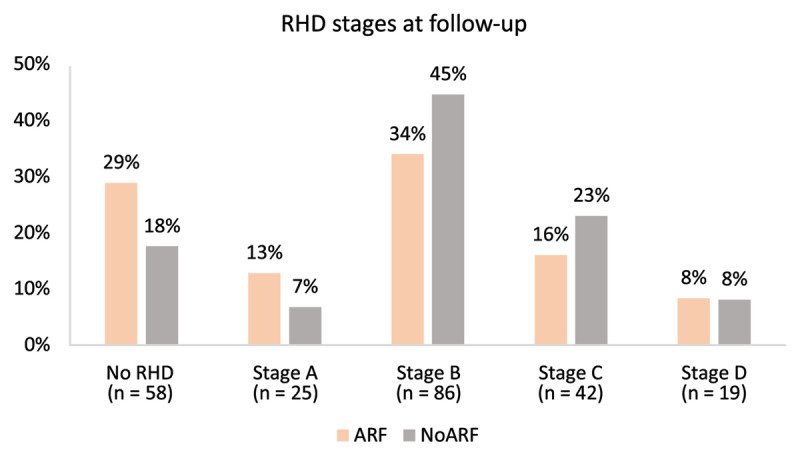
RHD stages at follow-up stratified by ARF and noARF status at baseline.

Two hundred and thirty (78.8%) individuals had follow-up data available (Table 3). There were differences in the proportion of RHD stages between the groups, notably more with Stage A in the ARF group and more with Stage B in the noARF group (*p* = 0.0078, Figure 3). There were also more cases of resolved/no RHD in the ARF group (28.8%) compared to the noARF group (17.6%); however, this did not reach statistical significance (*p* = 0.075). A comparison of early and advanced RHD showed no difference between the ARF status groups (*p* = 0.165).

**Table 3:** Summary of patient data RHD severity and lesion type at follow-up.


	ARF *n* = 156 *n* (%)*	No ARF *n* = 74 *n* (%)*	*P*-value^†^

Age at follow-up (years; mean (sd))*	15.4 (4.2)	16.8 (4.9)	0.031

Female	78 (50%)	50 (67.6%)	0.016

SAP adherence % (median, IQR) *n* = 197	81 (66–88)	82 (69–89)	0.966

***RHD stages***			0.008

Resolved RHD	45 (28.8%)	13 (17.6%)	

Early RHD – Stage A	20 (12.8%)	5 (7.1%)	

Early RHD – Stage B	53 (34.0%)	33 (44.6%)	

Advanced RHD – Stage C	25 (16.0%)	17 (23.0%)	

Advanced RHD – Stage D	13 (8.3%)	6 (8.1%)	

Total early RHD	73 (46.8%)	38 (51.4%)	0.165

Total advanced RHD	38 (24.4%)	23 (31.1%)	

Resolved RHD	45 (28.8%)	13 (17.6%)	

***Lesion type (resolved RHD excluded)***	*n* = 111	*n* = 61	

Pathological mitral regurgitation – all	92 (82.9%)	56 (91.8%)	0.166

Pathological aortic regurgitation – all	43 (38.7%)	16 (26.2%)	0.131

Isolated mitral regurgitation	11 (9.9%)	2 (3.3%)	0.141

Isolated aortic regurgitation	9 (8.1%)	3 (4.9%)	0.542

MR and AR (no abnormal morphology)	0 (0%)	1 (1.6%)	0.355

MR with abnormal morphology (+/– AV disease)	80 (72.1%)	53 (86.9%)	0.036

AR with abnormal morphology (+/– MV disease)	26 (23.4%)	10 (16.4%)	0.330

Any mitral stenosis	9 (8.1%)	3 (4.9%)	0.542

Any aortic stenosis	0 (0%)	0 (0%)	–


*Column percentage, unless otherwise specified. ^†^Fisher test, unless otherwise specified. AV, aortic valve; ARF, acute rheumatic fever; AR, aortic regurgitation; MR, mitral regurgitation; RHD, rheumatic heart disease, SAP, secondary antibiotic prophylaxis; sd, standard deviation.

### Lesion type at follow-up

Individuals with resolved RHD were excluded from the analysis of lesion type at follow-up. Pathological MR was again the most common lesion detected and was present in 82.9% of those in the ARF group and 91.8% in the noARF group (*p* = 0.1662). There was no difference in the proportions of *isolated* pathological MR (Stage A) with very few cases in either group (ARF: 11/111, noARF: 2/74, Table 3). Mitral regurgitation with morphological abnormalities (Stage B or greater) was present in 86.9% of those in the noARF group compared to 72.1% from the ARF group (*p* = 0.036).

### Change in RHD stage at follow-up

**Figure 4:**
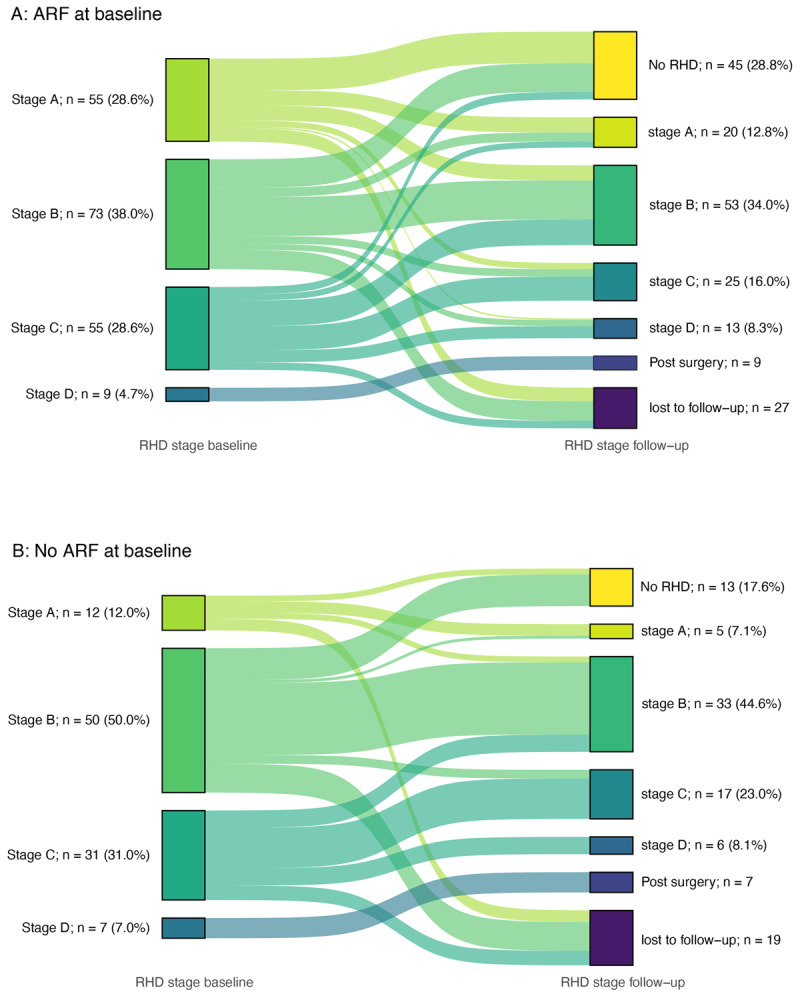
Sankey diagram demonstrating changes in RHD stage between baseline and follow-up echocardiogram. **4A** shows those with ARF at baseline. **4B** shows those with noARF at baseline. The width of each ribbon demonstrates the relative proportion of individuals in each stage at baseline and at follow-up.

Changes in RHD stage were more widespread in the ARF group, with some individuals progressing or regressing by multiple stages during follow-up (Figure 4A). The noARF group demonstrated more stable disease overall, with no individuals progressing by more than one disease stage (Figure 4B). Tabulated proportions are included in Supplement 3.

**Figure 5:**
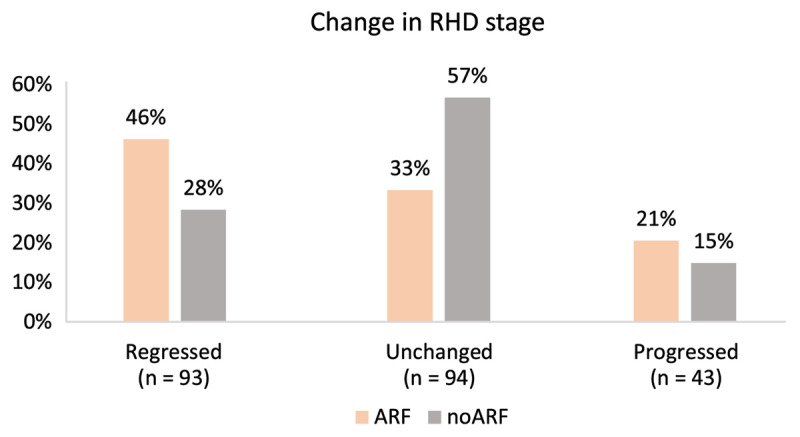
Progression/regression of RHD stage from baseline to end of follow-up period stratified by ARF status.

The relative difference in stability of RHD stage can be appreciated in Figure 5, with 57% unchanged in the noARF group compared to 33% unchanged in the ARF group.

## Discussion

Important findings from this study include that: i) a greater proportion of isolated regurgitation (Stage A) was seen in those with ARF at RHD diagnosis; ii) both groups included advanced disease and outcomes for those with Stage C RHD at baseline were the same for those with ARF and no ARF; and iii) valve disease was more likely to regress in severity in individuals with ARF at diagnosis compared to those with no ARF.

Secondary antibiotic prophylaxis is prescribed to all those diagnosed with ARF in Australia, and adequate cover can reduce ARF recurrences (12) and progression of RHD (13). We hypothesised that RHD severity may be worse in the noARF group given the lack of medical intervention following any undiagnosed Group A Streptococcus infection and/or ARF episode. We found a difference in the proportions of Stage A and Stage B RHD between the groups at diagnosis, with fewer morphological abnormalities (more Stage A) in the ARF group. Despite receiving an RHD diagnosis, this may represent individuals with acute valvulitis at diagnosis and explain the greater number demonstrating resolved RHD in the ARF group, with the valvulitis improving once the acute inflammatory response of ARF settled (14). Guidelines (5) recommend a diagnosis of ARF with carditis for those with valvulitis detected during an episode of ARF.

However, our findings highlight the difficulty in differentiating early RHD from acute valvulitis and the need for serial echocardiography in individuals with ARF. Due to diagnostic overlap between echocardiographic appearances of acute and chronic RHD and the high incidence of apparently missed ARF episodes, concurrent diagnosis of ARF and RHD frequently occurs in Australia. Improvements in health literacy and healthcare-seeking behaviour may improve ARF detection rates in Australian communities. These improvements may come from shifting the delivery of healthcare services to co-designed programs that prioritise the needs and voices of those that are most affected by RHD (15). Additionally, further refinement of the criteria used to diagnose ARF may improve detection rates. The Jones Criteria (10) are limited by their inability to provide a definitive ARF diagnosis. More sensitive testing could include the development of a simple blood test to provide a definitive diagnosis (16).

Individuals from the ARF group were younger at the time of RHD diagnosis. We also note that individuals demonstrating regression in RHD were younger than those who did not regress in severity. Further research is required to understand whether this difference reflects an association between age and RHD regression or can be attributed to the younger age of those with ARF at baseline.

Advanced RHD was present with or without ARF, and the proportion of Stage C disease progressing to surgery (Stage D) was high in both groups. This is an expected finding given the haemodynamic consequences of severe RHD, which dictate the need for surgical intervention (17) and aligns with previous studies demonstrating poor outcomes for those with advanced RHD at diagnosis (18).

There was no difference in the proportions of early and advanced RHD between the groups suggesting the probability of missed prior cases of ARF in both groups. This raises the possibility that RHD severity may not be linearly associated with time from initial ARF episode. This association cannot be tested since the date of any prior ARF episodes is unknown. Recent data suggests that febrile illness and echocardiographic evidence of carditis are the only indicators of ARF in some cases (19), further supporting the need for echocardiographic screening in high risk populations.

Limited resources in remote communities where the prevalence of RHD is high can restrict the timely delivery of follow-up cardiology services, including echocardiography. Therefore, for pragmatic reasons, a diagnosis of RHD following presentation with ARF is often made when valvular abnormalities are present.

Treatment is similar for those with a diagnosis of ARF with carditis and for RHD (5). However, there is evidence suggesting a label of RHD (even if it is mild) can impact quality of life (20,21). This must be weighed up against the potential risk of disease progression linked to reduced adherence to SAP for those diagnosed with ARF but not with significant RHD (22). Given the high number of individuals with Stage B or greater RHD in this cohort, early diagnostic labelling of RHD may be reasonable in those where follow-up echocardiography is expected to be difficult. The challenge of providing follow-up is reflected in the high number of individuals lost to follow-up in this cohort. Improved access to both ARF and RHD diagnosis and follow-up is required and could be addressed through echocardiographic task-sharing. This can include broadening the scope of practice for cardiac sonographers and the inclusion of briefly trained health practitioners to acquire echocardiographic screening images for interpretation by expert clinicians, which could be done remotely (23,24).

Valvular changes and regurgitation associated with early RHD can be subtle and might be considered as ‘trivial’ or ‘physiological’ in a low-risk population, particularly when an ARF diagnosis is absent. Our findings show, however, that regardless of ARF history, RHD may be present, and abnormalities were more likely to persist in those without ARF. While these findings may not be true in other populations, our data suggest that a lack of ARF diagnosis should not preclude a diagnosis of RHD in those from high-risk populations.

### Limitations

This study was performed retrospectively; therefore, some patients were excluded due to missing data. Original diagnostic reports were used in 5% of cases due to unavailability of echocardiographic images. These factors may reduce the accuracy of findings.

Application of the 2023 WHF guidelines was used to assign RHD stages to individuals obtained from the NT RHD register. The original diagnoses and entry to the register were based on previous guidelines (11), meaning it is possible that some cases of RHD were not included due to changes in the criteria for diagnosis. Specifically, this applies to young children who were previously classified with non-pathological MR based on the contemporaneous requirement of 2 cm MR jet length (11). Jet length requirements have been updated in the 2023 guidelines (9) to MR jet length of ≥1.5 cm in those < 10 years or weighing <30 kg.

It is possible that the RHD stage fluctuated throughout the follow-up period (in between the first and final echocardiograms). However, those changes have not been reported here. In this evaluation, Stage D RHD represents those having undergone surgical intervention, so it was not possible for those diagnosed with Stage D RHD to change stages.

These findings are from a population of young, predominantly Aboriginal Australians and may not reflect findings from other populations where RHD is endemic.

## Conclusions

In this Australian setting, diagnosis of RHD is made with or without evidence of ARF, and progression to advanced disease is possible in the absence of ARF diagnosis. These findings should provide confidence in the diagnosis and management of individuals with echocardiographic changes consistent with RHD in the absence of ARF diagnosis. Echocardiographic screening enables early detection of sub-clinical disease that may progress to advanced disease even in the absence of ARF at diagnosis and therefore offers opportunity to commence treatment and modify prognosis. Individuals who had no ARF at baseline were less likely to regress, further supporting raised awareness of the possible presence of RHD in high-risk Australian individuals and the need for early detection and treatment with SAP. This also supports the need for more sensitive testing for ARF in endemic populations to facilitate early commencement of SAP.

## Additional File

The additional file for this article can be found as follows:

10.5334/gh.1454.s1 Supplementary File. Supplement 1 to 3.
