# Supplementary material for: The Impact of Acute Rheumatic Fever Diagnosis on Rheumatic Heart Disease Severity
Source: Glob Heart. 2025 Aug 29;20(1):72. doi: 10.5334/gh.1454 (PMC12396188; doi:10.5334/gh.1454)
Supplement: Supplementary File. — Supplement 1 to 3. [file gh-20-1-1454-s1.pdf]

Supplement 1: Details of Valvular Lesion Severity Grading

Mitral regurgitation (MR) was classified as 'pathological' based on the 2023 World Heart Federation (WHF) guidelines for the diagnosis of RHD (9) detailed in Supplement 2. Severity of regurgitation was assessed in accordance with American Society of Echocardiography guidelines (7) through qualitative means based on the jet length, jet width, mitral inflow velocity (taking mitral stenosis into consideration), evidence of systolic flow reversal in pulmonary veins, spectral Doppler intensity, and left-sided chamber dilatation.

Aortic regurgitation (AR) was classified as pathological based on the 2023 WHF guidelines detailed in Supplement 2. Severity of AR was graded in accordance with American Society of Echocardiography guidelines (7) through qualitative means based on the jet length, jet width, left ventricular outflow velocity (LVOT obstruction excluded), evidence of diastolic flow reversal in the descending and abdominal aorta, spectral Doppler intensity, and left-ventricular chamber dilatation (considering the presence of significant mitral regurgitation).

Mitral stenosis (MS) was diagnosed based on the presence of morphological and functional mitral valve abnormalities, specifically leaflet thickening, commissural fusion, and restricted motion, in combination with the presence of an elevated mean trans-mitral pressure gradient in accordance with American Society of Echocardiography guidelines (8). In the absence of significant MR, trans-mitral pressure gradient (TMPG) $\geq$ 4mmHg was considered to represent mitral stenosis (9). In those with significant MR, the classification of stenosis was weighted more heavily on leaflet mobility and the mitral valve area which was calculated by two-dimensional planimetry or excluded via sufficient mitral leaflet separation (25).

Aortic stenosis (AS) was diagnosed by the presence of elevated mean aortic pressure gradient in association with reduced leaflet excursion (8).

Supplement 2: 2023 WHF Echocardiographic Criteria for Individuals Aged ≤20 Years

Definition of pathological regurgitation

**Pathological mitral regurgitation (all four Doppler echocardiographic criteria must be met)**
• Seen in two views
• In at least one view, jet length ≥2cm if ≥ 10 years, or ≥1.5cm if <10 years
• Velocity ≥3m/s for one complete envelope
• Pan-systolic jet in at least one envelope
**Pathological aortic regurgitation (all four Doppler echocardiographic criteria must be met)**
• Seen in two views
• In at least one view, jet length ≥1cm
• Velocity ≥3m/s in early diastole
• Pan-diastolic jet in at least one envelope

Morphological features of RHD

**Features in the MV**
• Anterior leaflet thickening (age-specific)* and/or chordal thickening
• Restricted anterior/posterior leaflet motion and/or excessive anterior leaflet tip motion
**Pathological aortic regurgitation (all four Doppler echocardiographic criteria must be met)**
• Cusp thickening
• Cusp prolapse
• Restricted cusp motion
• Coaptation defect in diastole

*≥3.0mm for individuals ≤20 years; ≥4.0mm for individuals 21-40 years; ≥5.0mm for individuals >40 years.

AR, aortic regurgitation; AV, aortic valve; MR, mitral regurgitation; MS, mitral stenosis; MV, Mitral valve; RHD, rheumatic heart disease; WHF, World Heart Federation.

Supplement 3: Change in RHD Stage Data

3A: ARF group data with baseline RHD stages and counts in columns one and two. Column percentages show proportion of each stage at baseline.

Follow-up RHD stages shown in columns 3-8 with cell count based on change from corresponding baseline RHD stage. Row percentages show proportion of relevant baseline stage.

		**Follow-up**					
**Baseline**		Resolved	Stage A	Stage B	Stage C	Stage D	Lost to follow-up
Stage A	55 (29%)	21 (38%)	10 (18%)	10 (18%)	4 (7%)	1 (2%)	9 (16%)
Stage B	73 (38%)	19 (26%)	6 (8%)	26 (36%)	5 (7%)	4 (5%)	13 (18%)
Stage C	55 (29%)	5 (9%)	4 (7%)	17 (31%)	16 (29%)	8 (15%)	5 (9%)
Stage D	9 (5%)	0 (0%)	0 (0%)	0 (0%)	0 (0%)	0 (0%)	0 (0%)

3B: No ARF group data with baseline RHD stages and counts in columns one and two. Column percentages show proportion of each stage at baseline.

Follow-up RHD stages shown in columns 3-8 with cell count based on change from corresponding baseline RHD stage. Row percentages show proportion of relevant baseline stage.

		**Follow-up**					
**Baseline**		Resolved	Stage A	Stage B	Stage C	Stage D	Lost to follow-up
Stage A	12 (12%)	2 (17%)	4 (33%)	2 (17%)	0 (0%)	0 (0%)	4 (33%)
Stage B	50 (50%)	11 (22%)	1 (2%)	25 (50%)	3 (6%)	0 (0%)	10 (20%)
Stage C	31(31%)	0 (0%)	0 (0%)	6 (19%)	14 (45%)	6 (19%)	5 (16%)
Stage D	7 (7%)	0 (0%)	0 (0%)	0 (0%)	0 (0%)	0 (0%)	0 (0%)

References

(7) Zoghbi WA, Adams D, Bonow RO, Enriquez-Sarano M, Foster E, Grayburn PA, et al. Recommendations for Noninvasive Evaluation of Native Valvular Regurgitation: A Report from the American Society of Echocardiography Developed in Collaboration with the Society for Cardiovascular Magnetic Resonance. *J Am Soc Echocardiogr*. 2017;30:303-371.
(8) Pandian NG, Kim JK, Arias-Godinez JA, Venkateshvaran, A, Campors Vieira LC, Little SH, et al. Recommendations for the use of echocardiography in the evaluation of rheumatic heart disease: A report from the American Society of Echocardiography. *J Am Soc Echocardiogr*. 2022;36:3-28.
(9) Rwebembera J, Marangou J, Mwita JC, Mocumbi AO, Mota C, Okello E, et al. 2023 World Heart Federation guidelines for the echocardiographic diagnosis of rheumatic heart disease. *Nat Rev Cardiol*. 2024;21:250-263.
(25) Williamson JM, Remenyi B, Horton AE, Morris P, Whalley GA. Mitral leaflet separation revisited: A surrogate of mitral valve area in young people with rheumatic mitral regurgitation living in remote areas. *J Am Soc Echocardiogr*. 2025; 38(1):49-50
